# Barbed suture *versus* preperitoneal ventral patch in medium-size ventral hernia repair: randomized clinical trial

**DOI:** 10.1093/bjsopen/zraf099

**Published:** 2025-10-31

**Authors:** Asmatullah Katawazai, Göran Wallin, Gabriel Sandblom

**Affiliations:** Department of Surgery, Faculty of Medicine and Health, Örebro University Hospital, School of Medical Sciences, Örebro University, Örebro, Sweden; Department of Surgery, Faculty of Medicine and Health, Örebro University Hospital, School of Medical Sciences, Örebro University, Örebro, Sweden; Department of Clinical Science and Education Södersjukhuset, Karolinska Institutet, Stockholm, Sweden

**Keywords:** umbilical hernia, epigastric hernia, linea alba

## Abstract

**Background:**

This study aimed to compare preperitoneal ventral mesh patch with barbed suture in ventral hernia repair, evaluating recurrence rates and complications, and to assess the safety of preperitoneal patch placement.

**Methods:**

In this randomized clinical trial, adult patients undergoing ventral hernia repair at Karlskoga Hospital between 2020 and 2023 were randomized 1 : 1 to either a ventral mesh patch repair group or a non-absorbable barbed suture repair group, blinded to patients and outcome assessors. The primary outcome was recurrence detected at clinical examination and CT verification 1 year after surgery. Pain (measured on a visual analogue scale and using the Ventral Hernia Pain Questionnaire), nausea, and surgical site events (including wound infection, haematoma and seroma) were assessed 4 h, 1 week, 1 month, and 4 years after operation.

**Results:**

Of 256 eligible patients, 209 were screened, and 205 were randomized to ventral mesh patch repair (103) or barbed suture repair (102). The hernia recurrence rate at 1 year was lower in the ventral patch repair group (1.9 *versus* 5.9%), although this was not statistically significant (*P* = 0.14). The surgical site infection rate at 1 month was significantly lower in the ventral patch group (0.9 *versus* 6.9%; *P* = 0.02). At 1 month, the ventral patch repair group had higher ‘pain right now’ scores on the Ventral Hernia Pain Questionnaire (*P* = 0.02), although this difference had disappeared by 1 year.

**Conclusion:**

Preperitoneal ventral hernia patch repair is a safe and effective technique with a recurrence rate not statistically significant from that after barbed suture repair. Although postoperative pain scores at 1 month were higher after ventral patch repair, this difference had disappeared by 1 year.

## Teaser

This randomized clinical trial compared the safety and effectiveness of a preperitoneal ventral hernia patch with a non-absorbable barbed suture repair (without mesh) in patients with medium-sized primary ventral hernias. Although the preperitoneal patch group experienced fewer recurrences, the difference was not statistically significant, and both methods demonstrated acceptable safety profiles.

## Introduction

Primary ventral hernias (PVHs), including umbilical and epigastric hernias, are common and frequently require surgical intervention. In an American hospital-based study involving 302 individuals who underwent ultrasound imaging for reasons other than hernia, it was found that an asymptomatic ventral hernia was present in approximately 25% of women and 23% of men^1^. The median age for women with a symptomatic ventral hernia is 31–40 years, whereas for men it is 61–70 years^2^. The optimal surgical method of repair remains controversial. Various techniques are available, including mesh reinforcement and suture repair^3,4^.

The pathogenesis of PVH is not fully understood^5^, although it is probably a multifactorial disease involving both endogenous and exogenous factors. Abnormal collagen composition is considered a significant contributor to abdominal wall weakness and hernia formation^6^. Hernias are also associated with conditions that increase intra-abdominal pressure or weaken the abdominal wall, such as truncal obesity, pregnancy, ascites, and rectus diastasis. Increased intra-abdominal pressure exerts a mechanical force on abdominal contents, causing them to protrude through the hernia defect, exacerbating symptoms by increasing tissue tension, possibly leading to ischaemia^6–9^. Furthermore, the presence of increased intra-abdominal and subcutaneous fat complicates repair, and increases the risk of postoperative complications such as seroma, bleeding, and surgical site infection (SSI)^10^.

Some surgeons recommend elective repair of PVH upon diagnosis, whereas others advocate watchful waiting in patients with asymptomatic hernia^7^. Mesh-reinforced or suture repairs are used in PVH repair^11^. However, for umbilical hernias up to 1 cm there is little evidence supporting the superiority of mesh repair. A meta-analysis found that the recurrence rate after mesh repair was slightly less than that after polypropylene suture repair without mesh in small hernia repair^3^. Although recent studies have demonstrated the superiority of mesh repair for medium and small ventral hernias, comparison with barbed suture repair has yet to be undertaken.

Mesh implantation may lead to persistent pain or other mesh-related complications^12^. In a large database study of ventral hernia, the use of mesh was associated with an increased readmission rate. Open surgical repair is common for medium-sized hernias (1–4 cm)^13^. Despite the widespread use of composite ventral mesh patches, few studies have evaluated their advantages and disadvantages. Ventral patches are usually placed intraperitoneally; preperitoneal placement has not yet been studied^14,15^.

In recent years, barbed suture has been used widely in the repair of umbilical and incisional hernias, both laparoscopically and in open surgery^16^. However, no study has compared ventral patch with non-absorbable barbed suture in open PVH repair.

A ventral composite mesh was placed in a preperitoneal position to avoid mesh contact with content of the abdominal cavity and reduce the risk of severe complications. This study compared the use of preperitoneal ventral mesh patch with non-absorbable barbed suture repair in ventral hernia surgery, investigating recurrence rates and short- and long-term postoperative complication rates associated with these two methods.

Studies of preperitoneal placement of a ventral patch are sparse in the literature. Intraperitoneal placement is questioned owing to potential risks including fistula formation and adhesions, if not placed correctly^17^. This trial aimed to assess the safety and efficacy of preperitoneal placement in PVH repair, and also evaluated and compared the non-absorbable barbed suture in terms of recurrence rate.

## Methods

### Trial design

This single-centre, double-blind, parallel-group, randomized clinical trial (RCT) was conducted at Karlskoga Hospital in the County of Örebro, Sweden. Eligible patients were identified and recruited at the Hernia Centre, Karlskoga Hospital. Patients had applied for surgery directly or been referred from district healthcare clinics or other hospitals. All patients received written and verbal information about the study, and those who agreed to participate signed an informed consent form before the intervention. Randomized allocation occurred in the operating theatre after the patient had been anaesthetized and before the start of surgery; the patient was then officially included. The study was approved by the Swedish Ethics Review Authority (2020-02173) and registered at ClinicalTrials.gov (NCT04356976). This RCT adhered to the CONSORT recommendations.

### Participation and eligibility criteria

Inclusion criteria were adults aged > 18 years with a PVH (umbilical or epigastric) with a defect size ranging from 1 to 4 cm, determined by physical examination, and a body mass index (BMI) of less than 35 kg/m². Exclusion criteria were recurrent hernia, pregnancy, poor knowledge of the Swedish language, and dementia.

Baseline demographic information and full medical histories were registered at the preoperative clinical visit. A standardized physical examination was conducted, and pain was assessed using a visual analogue scale (VAS). Additional data, such as co-morbidities (including smoking status, medications, previous abdominal operations, BMI), waist circumference, hernia defect size (measured during surgery) and American Society of Anesthesiologists (ASA) grade were recorded, along with measures of the outcomes of interest (see below).

In elective cases, patients were offered participation in the trial at the time of first referral to the hernia centre, when the surgeon scheduled the patient for surgery. In acute or subacute cases, patients were offered participation when the surgeon scheduled them for surgery.

All patients who met the inclusion criteria and provided informed consent were considered eligible for inclusion. Randomization was performed intraoperatively after the patient was prepped for surgery and had received general anesthesia. Therefore, the modified intention-to-treat (mITT) population consisted of all randomized patients who underwent surgical intervention according to their assigned group.

### Intervention

All repairs were performed at the hernia centre by experienced surgeons familiar with both techniques. Patients received single-dose antibiotic prophylaxis immediately before surgery according to the authors’ routine protocol. After revelation of the randomized technique to be used, the surgeon operated accordingly, but the patients were not informed of the technique used.

For ventral patch repair, after reducing the hernia sac, the peritoneum was gently dissected from the abdominal wall to create room for the hernia patch. A round composite mesh patch (Ventralex™; Bard Franklin Lakes, NJ, USA) was placed in the preperitoneal space. Mesh size was selected based on defect size: a 6.4-cm diameter mesh for defects of 1–2 cm and an 8-cm diameter mesh for defects of 2–4 cm. Mesh straps were fixed to the fascia with single non-absorbable 2/0 monofilament sutures as recommended. The fascial defect was then closed horizontally with a continuous 2/0 Prolene™ suture (Ethicon, Cincinnati, OH, USA).

For barbed suture repair, after reducing the hernia sac, the hernia defect was closed horizontally using a non-absorbable, unidirectional 2/0 Stratafix™ barbed suture (Stratafix™ 2/0 polypropylene spiral; Ethicon) with a standard 4 : 1 small-bite closure. No mesh was used in this group.

In both groups, subcutaneous tissue was sutured with a 2/0 Vicryl™ suture (Ethicon), and the skin was closed intracutaneously with a continuous 3/0 Monocryl™ suture (Ethicon).

### Randomization

The randomization sequence was created by the Research Infrastructure Office at Region Örebro County using computer-generated variable block randomization. Patients were randomized in a 1 : 1 ratio to either the ventral patch repair group or the barbed suture repair group, and stratified to ensure equal distribution between groups. Randomization was undertaken shortly after patients had been anaesthetized on the operating table when operating theatre staff retrieved and opened the opaque sealed envelope, revealing the group allocation.

### Blinding

Recruiting and operating surgeons were not involved in the randomization process. Because surgery was conducted under general anaesthesia, patients were unaware of their group allocation. Outcome assessors and the rest of the research team were also blinded to the group allocation. At each follow-up visit, a physician blinded to the procedure conducted a clinical examination.

### Outcomes of interest

The primary outcome was hernia recurrence 1 year after operation, assessed by clinical examination. If recurrence was suspected clinically, computed tomography (CT) was performed to confirm the diagnosis and compare outcomes between the two groups.

Secondary outcomes included intraoperative and postoperative complications, including SSIs, based on clinical examination, as well as surgical site events (SSEs), reoperations, and emergency department visits. Postoperative pain was assessed using both a VAS and the Ventral Hernia Pain Questionnaire (VHPQ).

Short-term postoperative data focused on patient-reported outcomes including pain, nausea, and SSEs. These were recorded at 4 h, 1 week, 1 month, and 1 year after operation. At 4 h and 1 week after surgery, assessments were conducted by an experienced nurse at the hernia centre via video follow-up using the Visiba Care application. If any signs or symptoms of complications such as seroma, haematoma, infection, or wound dehiscence were noted, the patient was promptly scheduled for an in-person evaluation with a specialist surgeon within 1 week. During the video follow-ups, wound healing and any short-term complications were documented systematically.

At 1 month and 1 year after surgery, patients were examined by an experienced specialist in abdominal wall surgery in day clinic. Any sign of complication, such as seroma, haematoma, wound dehiscence, or suspected hernia recurrence, was recorded. If a complication was suspected at follow-up, the patient underwent ultrasound examination or CT to confirm that suspicion. Wound complications and issues related to hernia repair were documented. If hernia recurrence was suspected, the patient underwent CT to confirm or exclude recurrence or other complication. Clinical records were reviewed to identify events between scheduled visits, and patients were asked to report any events related to the operation. During these follow-up visits, patients completed the VHPQ, an 18-question survey designed to assess pain and discomfort related to ventral hernias and their repair. The VHPQ explores different domains of quality of life, including physical activity and social aspects^12^. All patient data were collected prospectively during the hospital stay and at outpatient visits, according to the study protocol, and registered in the Greenlight Guru Clinical Database. The database was provided free of charge to the author by Region Örebro County, Sweden.

### Sample size

The study was powered to detect the primary endpoint, a clinically and CT-verified hernia recurrence 1 year after surgery. According to the European Hernia Society guidelines published in 2014, suture repair may have recurrence rates of up to 20%, whereas mesh repair significantly reduces this risk. It was planned to include a total of 200 patients, assuming a 20% cumulative recurrence rate without mesh repair and a 3% rate with ventral patch repair. Based on these assumptions, a two-sided test with an α level of 0.05 and 80% power would require 176 patients. To account for potential drop-outs, it was aimed to include 200 patients. However, owing to the long waiting list for elective repair, many patients were excluded before the intervention. Some chose to have their hernia repaired elsewhere, whereas others developed an illness that made them unwilling or unable to undergo the planned surgery. To maintain the study’s statistical power, approval was obtained from the Swedish Ethics Review Authority (2023-03503-02) to include an additional 20 patients.

### Statistical analysis

All data were recorded electronically and analysed using SPSS version 29.0 (IBM, Armonk, NY, USA). Continuous variables were expressed as mean(standard deviation, s.d.) and evaluated using Student’s *t* test. Categorical variables, such as recurrence rate and complication rate, were compared by means of the χ^2^ test. Analyses stratified for defect size were made for recurrence rate and pain rating. The hernias were categorized depending on defect diameter into groups with a defect of 1–2, 2–3, and > 3 cm. Pain scores from the VHPQ were analysed using the Mann–Whitney *U* test owing to their skewed distribution. *P* ≤ 0.050 was considered statistically significant. A sensitivity analysis was conducted comparing intention-to-treat (ITT) analyses, in which individuals were kept in their originally assigned groups where feasible, and per-protocol analyses that excluded them. The recurrence and complication rates were similar under both approaches, suggesting that these exclusions did not materially alter the primary conclusions.

## Results

### Participants

**Fig. 1:**
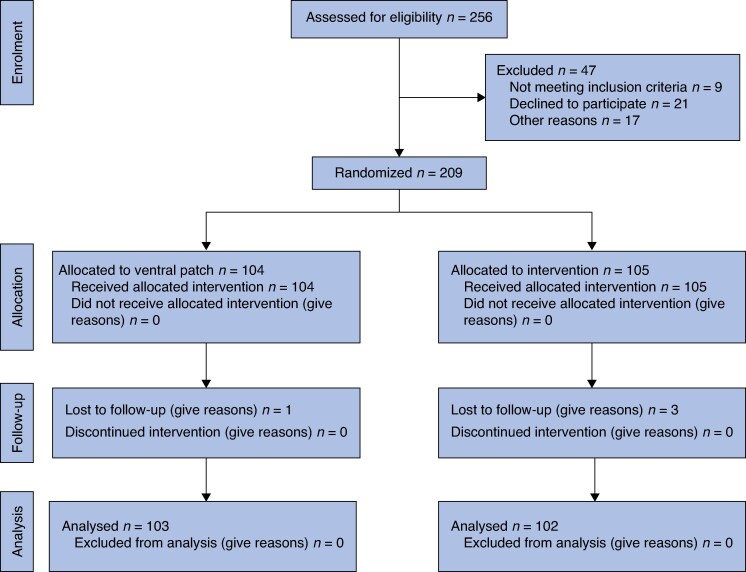
CONSORT diagram for the trial

A total of 256 consecutive eligible patients were assessed for inclusion. Forty-seven patients were excluded before hernia repair surgery for the following reasons: did not meet inclusion criteria (9); refused to participate (21); sought repair elsewhere (10); too ill for surgery (5); and underwent emergency surgery (2). One patient on the waiting list underwent emergency surgery at another hospital for hernia strangulation that required bowel resection and primary anastomosis. This patient developed anastomotic failure 2 days later and subsequently died at the same hospital following complications. Consequently, 209 patients were randomized, with 104 in the ventral patch repair group and 105 in the barbed suture group. Four patients (1 in the ventral patch repair group and 3 in the barbed suture repair group) were excluded after surgery: three declined to attend scheduled follow-ups, and one underwent surgery for colorectal cancer, resulting in 205 patients for final analysis. Of the four patients excluded, two declined to attend the 1-year follow-up but had no documented wound or hernia-related visits in the electronic medical records; one patient had moved to another city; and another declined the follow-up because of travel distance. The final 1-year analysis was therefore conducted on 205 patients according to a modified ITT principle, including all randomized and operated patients with 1-year follow-up data (*Fig. 1*).

### Baseline data and patient characteristics

A total of 205 patients, 158 men (77%) and 47 women (23%), were analysed; 103 were allocated to the ventral patch repair group and 102 to the barbed suture repair group. The mean(s.d.) age was 61(13.5) years overall, 58.1(15.2) years among women and 62(13.0) years among men. The mean(s.d.) BMI was 28.2(3.6) kg/m² (*Table 1*).

**Table 1:** Patient characteristics

	Barbed suture (*n* = 102)	Ventral patch (*n* = 103)
**Sex**		
Female	29 (28.4%)	18 (17.5%)
Male	73 (71.6%)	85 (82.5%)
Diabetes	10 (9.8%)	10 (9.7%)
Cardiovascular disease	19 (18.6%)	13 (12.6%)
Anticoagulant use	5 (4.9%)	7 (6.8%)
**ASA fitness grade**		
I	31 (30.4%)	35 (33.9%)
II	54 (52.9%)	48 (46.6%)
III	16 (15.7%)	19 (18.5%)
IV	1 (1.0%)	1 (1.0%)
**Smoking status**		
Never smoked	67 (65.7%)	60 (58.3%)
Quit smoking	25 (24.5%)	30 (29.1%)
Smoker	6 (5.9%)	5 (4.9%)
Occasional smoker	3 (2.9%)	6 (5.8%)
Other/unknown	1 (1.0%)	2 (1.9%)
Age, years, mean(s.d.) (s.e.)	61.9(13.3) (1.3)	60.7(13.9) (1.3)
BMI, kg/m^2^, mean(s.d.) (s.e.)	28.0(3.6) (0.3)	28.4(3.6) (0.3)
Waist measurement, cm, mean(s.d.) (s.e.)	102.8(13.2) (1.3)	103.9(12.7) (1.2)
Hernia defect size, cm, mean(s.d.) (s.e.)	2.0(0.7) (0.0)	1.9(0.5) (0.0)
Preoperative pain score, 0–10 VAS, mean(s.d.) (s.e.)	5.4(2.4) (0.2)	4.9(2.5) (0.2)

Values are *n* (%) unless stated otherwise. ASA, American Society of Anesthesiologists; s.d., Standard deviation; s.e., standard error; BMI, body mass index; VAS, visual analogue scale.

Most patients underwent elective repair (177, 87.2%); the remainder underwent subacute (16, 6.9%) or acute (12, 5.9%) procedures. Most patients had an ASA grade or I or II (82%), whereas 18% had grade of III–IV. The mean(s.d.) hernia defect size was 1.9(0.6) cm in the ventral patch repair group and 2.0(0.7) cm in the barbed suture group.

Statistical analyses were used to assess potential confounding variables between the two groups, including ASA grade, BMI, waist measurement, smoking status, anticoagulant use, and age. Levene’s test for equality of variances indicated equal variances for all variables (*P* > 0.050), allowing the use of standard *t* tests. Independent-samples *t* tests revealed no statistically significant differences between the groups for any of these variables (*P* ≥ 0.314). χ^2^ tests for diabetes, cardiovascular disease, kidney failure, liver disease, other diseases, and anticoagulant use also showed no significant associations. These findings suggest that the groups were comparable with respect to these variables, reducing the likelihood of confounding (*Table 1*).

### Outcomes and estimation

Hernia recurrence at 1 year was observed in six patients (5.9%) in the suture repair group and two (1.9%) in the ventral patch repair group (*P* = 0.14) (*Table 2*). No differences were found in analyses stratifying for defect diameter. Waist circumference was not associated with recurrence (*P* = 0.5).

**Table 2:** Summary of postoperative results

	Barbed suture (*n* = 102)	Ventral patch (*n* = 103)	P*
Pain score at 4 h, mean	1.2	2.2	0.001†
Nausea at 4 h	8 (7.9%)	5 (5.1%)	0.483
Nausea at 1 week	4 (3.9%)	8 (7.8%)	0.204
Surgical site infection at 1 week	1 (0.9%)	2 (6.9%)	0.559
Surgical site infection at 1 month	7 (6.9%)	1 (0.9%)	0.020
Seroma	2 (1.9%)	7 (6.9%)	0.080
Hernia recurrence at 1 year	6 (5.9%)	2 (1.9%)	0.141
Patient satisfaction at 1 year (% satisfied)	92.1	93.1	0.790
VHPQ ‘pain right now’ at 1 month	Low intensity	High intensity	0.020†
VHPQ rating at 1 year	Low pain intensity	Low pain intensity	0.760†

Values are *n* (%) unless stated otherwise. VHPQ, ventral Hernia Pain Questionnaire. *χ^2^ test, except †independent samples *t*-test.

Smoking was the only variable with a statistically significant association with recurrence (*P* < 0.001), suggesting that smoking status may be a key risk factor independent of the chosen repair technique.

There were no statistically significant differences in age, BMI, waist circumference, hernia size, and SSI rates between groups with and without recurrence, although there was a non-significant trend toward a larger defect size in the recurrence group.

At the 4-h follow-up, eight patients (7.9%) in the barbed suture group and five (5.1%) in the ventral patch repair group reported nausea (*P* = 0.483) (*Table 2*). At the 1-week follow-up, four patients (3.9%) in the barbed suture group and eight (7.8%) in the ventral patch repair group reported nausea (*P* = 0.2).

At 4 h after surgery, the pain score was significantly higher in the ventral patch repair group compared with the barbed suture group (mean VAS 2.2 *versus* 1.2; *P* = 0.001). At the 1-week follow-up, the pain score remained higher in the ventral patch repair group, but the difference was not statistically significant (*P* = 0.07). The mean(s.d.) VAS score was 5.1(2.5) 1 year after the repair. Among all patients, 63.6% reported pain that could not be ignored, whereas 36.4% experienced no pain or pain that was easily ignored.

At 1 month after operation, SSI was observed in seven patients (6.9%) in the suture repair group and one (0.9%) in the ventral patch repair group (*P* = 0.02). No SSI was reported in either group at the 1-year follow-up.

Seroma occurred in two patients (1.9%) in the barbed suture group and seven (6.9%) in the ventral patch repair group (*P* = 0.08).

At 1 month after surgery, patients in the ventral patch repair group registered higher pain intensity and greater interference with daily activities on the ‘pain right now’ item compared with the suture repair group (*P* = 0.02). There was no significant difference between the groups in the ‘worst pain in the preceding week’ rating. At 1 year, no significant differences were found between the groups in any of the VHPQ ratings.

At 1 year after surgery, 92.1% of patients in the barbed suture group and 93.1% in the ventral patch repair group reported being satisfied with the hernia repair (not statistically significant).

### Subgroup analyses

A subgroup analysis based on hernia defect size (< 2 *versus* ≥ 2 cm) revealed no significant differences in recurrence rates (*Table S1*); all procedures were performed by experienced surgeons, minimizing variability in surgical skill. A multivariate logistic regression model, including age, BMI, waist circumference, hernia defect size, and postoperative infection, showed no statistically significant predictors of recurrence (overall *P* = 0.611), likely owing to the low recurrence rate (8 of 205). However, the observed difference in recurrence (1.9% *versus* 5.9%) may still hold clinical relevance, warranting further investigation with larger samples and broader data collection.

Of note, patients were subgrouped according to waist circumference into those with a circumference of < 90, 90–109, and ≥ 110 cm. In the first subgroup, 15.8% of patients treated with a barbed suture had a recurrence, whereas none of the patients treated with a ventral patch reported recurrence. In the second subgroup the difference was 3.8 *versus* 1.9%, and in the third subgroup it was 3.2 *versus* 2.7% (*Table S2*).

Smoking was a strong predictor in univariable analysis (*P* = 0.002) but became borderline significant in the multivariate model (*P* = 0.072). One of the categories was significantly associated with recurrence (odds ratio 13.8; *P* = 0.04). In a separate analysis the association between smoking and recurrence was strengthened, there being a significantly higher prevalence among smokers (Pearson χ² = 17.74; *P* < 0.001) (*Table S3*). These findings suggest that smoking is a plausible risk factor for recurrence, requiring further validation in future studies with more events and simplified smoking classifications.

## Discussion

The present study has shown that hernia repair using a ventral patch is acceptably safe when the mesh is placed in the preperitoneal space. Preperitoneal placement of a ventral hernia patch resulted in significantly fewer SSIs than with suture repair 1 month after surgery. Patients undergoing ventral patch repair reported higher immediate postoperative pain, but this difference did not persist at 1 year. The recurrence rate was lower in the ventral patch repair group, although this was not statistically significant. Ventral patch repair is associated with lower SSI rates and does not lead to increased long-term pain or recurrence.

The median hernia defect size in the barbed suture group was slightly greater than that in the patch repair group, but without statistical significance. The recurrence rate 1 year after surgery was lower in the ventral patch repair group, although this difference was not statistically significant. Patients in the ventral patch repair group reported slightly higher pain levels during the first month after repair, but this difference did not persist at the 1-year follow-up. These findings are in line with previous literature comparing suture repair and ventral hernia patch methods for PVHs. Earlier studies reported that the use of mesh or ventral hernia patch repairs is associated with reduced long-term recurrence rates and durable outcomes, albeit sometimes accompanied by transient increases in early postoperative pain and discomfort. Several studies have examined the outcomes of open mesh repair for small and medium-sized PVHs, and consistently demonstrated a lower recurrence rate than with suture-only repair. In a RCT^18^ comparing suture repair with mesh repair for umbilical hernias smaller than 3 cm, recurrence rates were notably higher in the barbed suture group (approximately 11%), compared with only 1% among those receiving mesh. A subsequent systematic review and meta-analysis^19^ replicated these findings, showing that mesh-based repairs of small-to-medium umbilical hernias had consistently lower recurrence rates without a significant increase in complications.

This study has limitations. First, a 1-year follow-up period is short when evaluating long-term hernia recurrence rates, as recurrence is known to increase with time. A 3-year follow-up study to better assess long-term differences between the two groups is planned. Second, there were missing data on four patients who declined to attend scheduled visits. This could have affected the results because the patients were not equally distributed between the groups. Third, although patients showing signs of a complication were examined with CT, it is possible that a clinically undetectable hernia recurrence may have been missed. Finally, external factors such as the COVID-19 pandemic and limited operating theatre resources led to a long waiting list for hernia repair. This led to some patients seeking hernia repair elsewhere or postponing surgery owing to intervening co-morbidity, reflecting the real-world challenges facing many hospitals in Sweden.

Despite specific local circumstances and delays in hernia repair, the study results were relatively similar to those of previous research on mesh placement and suture repair in general. Of note is that there were no serious complications in the preperitoneal ventral patch repair group. However, studies of barbed suture repair as well as preperitoneal mesh placement are lacking, so caution should be observed when applying the findings of this study to broader populations.

This RCT compared preperitoneal ventral patch repair and barbed suture repair for ventral hernia repair. The rate of hernia recurrence, the primary outcome, was lower in the ventral patch repair group, although the difference was not statistically significant. There were no significant differences in secondary outcomes between the groups 1 year after surgery.

Previous studies and guidelines have suggested that suture repair leads to higher recurrence rates than mesh repair, with some meta-analyses reporting recurrence rates as high as 20% within 1 year for suture repairs^20^. In this study, the higher recurrence rate in the barbed suture group mirrors previous findings, although the difference was not statistically significant, possibly owing to better performance of the barbed suture. The findings indicate that preperitoneal placement of a ventral patch is a safe and effective method with no serious short- or long-term complications, supporting the other studies that reported low recurrence rates and high patient satisfaction with ventral patch repair.

## Supplementary Material

zraf099_Supplementary_Data

## Data Availability

The data that support the findings of this study are available on request from the corresponding author.
