# Supplementary material for: Barbed suture versus preperitoneal ventral patch in medium-size ventral hernia repair: randomized clinical trial
Source: BJS Open. 2025 Oct 31;9(6):zraf099. doi: 10.1093/bjsopen/zraf099 (PMC12578299; doi:10.1093/bjsopen/zraf099)
Supplement: zraf099_Supplementary_Data [file zraf099_supplementary_data.docx]

**Barbed Suture vs Preperitoneal Ventral Patch in Medium Size Ventral Hernia Repair: A Randomised Control Trial**

**Authors**

Asmatullah Katawazai^1^

Göran Wallin^1^

Gabriel Sandblom^2^

^1^ Department of Surgery, Faculty of Medicine and Health, Örebro University Hospital, School of Medical Sciences, Örebro University, Sweden

^2^ Department of Clinical Science and Education Södersjukhuset, Karolinska Institute, Stockholm, Department of Surgery, Södersjukhuset, Stockholm, Sweden

**Corresponding author:**

Asmatullah Katawazai

Department of Surgery

Örebro University Hospital

Karlskoga Lasarett, 691 44 Karlskoga

**ORCID ID** 0000-0002-3603-5606

**Supplementary Materials - Index**

| **Supplementary Results** |  |
| --- | --- |
| Supplementary Table 1 | *pag. 2* |
| Supplementary Table 2 | *pag. 2* |
| Supplementary Table 3 | *pag. 2* |

**Supplementary Results**

**Table S1. Defect diameter and recurrence rate.**

|  | Barbed Suture | Ventral patch | All patients |
| --- | --- | --- | --- |
| ≤2 cm | 4/67 (6.0%) | 1/80 (1.3%) | 5/147 (3.4%) |
| >2 cm | 2/35 (5.7%) | 1/23 (4.3%) | 3/58 (5.2%) |

**Table S2. Waist circumference and recurrence rate.**

|  | Barbed Suture | Ventral patch | All patients |
| --- | --- | --- | --- |
| <90 cm | 3/19 (15.8%) | 0/14 (0.0%) | 3/33 (9.1%) |
| 90–109 cm | 2/52 (3.8%) | 1/52 (1.9%) | 3/104 (2.9%) |
| ≥110 cm | 1/31 (3.2%) | 1/37 (2.7%) | 2/68 (2.9%) |

**Table S3. Recurrence vs risk factors**

|  | Recurrence (n) | No recurrence (n) | P-Value |
| --- | --- | --- | --- |
| Smoking | 8 | 197 | <0.001 |
| Waist circumference | 8 | 197 | 0.565 |
| Age | 8 | 197 | 0.528 |
| BMI | 8 | 197 | 0.353 |
| SSI | 8 | 197 | 0.110 |
| Hernia size | 8 | 197 | 0.555 |
